# Impaired retinoic acid receptor-γ signaling underlies a heritable form of urothelial keratinizing squamous metaplasia

**DOI:** 10.1016/j.xhgg.2026.100590

**Published:** 2026-03-13

**Authors:** Kaya Fukushima, Nicole Avery, Jade Desjardins, Benjamin J. Halliday, Zandra A. Jenkins, Robert Porteous, Tim Morgan, Padmini Parthasarathy, Michael Lau, Michael W. Vincent, Karen J. Liu, Stephen R.F. Twigg, Stephen P. Robertson

**Affiliations:** 1 Department of Paediatrics and Child Health, Dunedin School of Medicine, University of Otago, Dunedin 9016, New Zealand; 2 Department of Urology, Dunedin Hospital, Dunedin 9016, New Zealand; 3 Centre for Craniofacial and Regenerative Biology, King’s College London, SE1 9RT London, UK; 4 MRC National Mouse Genetics Network, Congenital Anomalies Cluster, Harwell, OX11 ORD Oxfordshire, UK; 5 Department of Pathology, Dunedin School of Medicine, University of Otago, Dunedin 9016, New Zealand; 6 Awanui Laboratories, Dunedin 9016, New Zealand; 7 Department of Urology, Southland Hospital, Invercargill 9812, New Zealand; 8 MRC Weatherall Institute of Molecular Medicine, John Radcliffe Hospital, University of Oxford, OX3 9DS Oxford, UK

**Keywords:** keratinizing desquamative squamous metaplasia, retinoic acid receptor gamma

## Abstract

Keratinizing desquamative squamous metaplasia (KDSM) of the urinary tract is typically a sporadic condition with unclear etiology and treatment options. It is characterized by either a focal or widespread transition of normal urothelium of the bladder and ureters to a stratified squamous keratinizing epithelium. Four individuals from three generations of a single family were ascertained with a likely autosomal dominant form of syndromic KDSM. Whole-genome sequencing was performed on three affected individuals and a truncating variant (*RARG* NM_000966.6:c.1237C>T; NP_000957.1:p.Arg413∗) in the gene encoding retinoic acid receptor gamma (RARγ) was identified to be segregating with the phenotype. The truncating variant does not destabilize the transcript or protein produced from this allele but instead predicts the loss of half of helix 12 of RARγ, leading to reduced responsiveness of the receptor to all-*trans* retinoic acid via a dominant-negative mechanism. Mice heterozygous for the variant demonstrated upregulation of cytokeratin-10 in the bladder and ureteric epithelium consistent with keratinizing squamous metaplasia of the urothelium. The implicated dominant-negative mechanism reduces retinoic acid signaling via heterodimeric receptors that incorporate the variant γ subunit and indicates that this condition may be addressable with high-dose retinoic acid receptor agonists.

## Teaser

A truncating variant in *RARG*, a gene previously not associated with monogenic human disease, segregates in a family with a heritable form of urothelial keratinizing squamous metaplasia. This genetic variant reduces retinoic acid signaling and suggests that deficient retinoic acid function could underlie sporadic and genetic forms of the condition.

## Introduction

Retinoic acid (RA) signaling is widely deployed across development.^1^^,^^2^ A deficiency of vitamin A, the precursor for RA, results in disorders of epithelia^3^ and reflective of this role in epithelial development and maintenance, RA analogs are used therapeutically to treat disorders of epithelia by influencing cellular differentiation.^4^^,^^5^^,^^6^

RA exerts its biochemical effects through binding to cytoplasmic receptors that are composed of heterodimers of either one of RARα, RARβ, or RARγ in conjunction with one of three RXR isoforms. Engagement of RA with these receptors results in the translocation of the complex to the nucleus and the activation of various transcriptional programs by binding to specific sequences (RA response elements [RAREs]) within gene promoters and enhancers.^7^ Diminished or insufficient signaling via RA receptors lead to a variety of disorders including a monogenic disorder characterized by microphthalmia (MCOPS12, OMIM: 615524), caused by truncating variants in *RARB*.^8^

Keratinizing desquamative squamous metaplasia (KDSM) of the urinary tract is a rare, typically sporadic condition that is characterized by the transition of normal urothelium to a stratified squamous keratinizing epithelium.^9^ Keratinized cellular debris typically accumulates over time within the bladder and ureter manifesting clinically with lower urinary tract symptoms including urgency, frequency, ureteric obstruction, and occasionally hematuria.^9^ Most reported cases have been sporadic with no clear etiology identified. Chronic inflammation, irritation, and micronutrient deficiencies are all possible factors contributing to the pathogenesis of the condition.^10^^,^^11^ Genetic factors have also been suggested based on the occurrence of KDSM in a single family, consistent with autosomal dominant inheritance of the trait.^9^^,^^12^ Several reports have described instances where vitamin A supplementation was successful in treating KDSM in the context of vitamin A deficiency.^10^^,^^11^ Consistent with a possible link between KDSM and RA signaling, vitamin A deficiency in murine models reproduces a phenotype similar to KDSM.^3^^,^^13^^,^^14^^,^^15^

Recently, we reported a family of four individuals (three females, one male) from three generations affected by a syndromic form of KDSM.^16^ In addition to urothelial metaplasia, they also reported dry mouth, dry eyes, and manifested mild short stature, suggestive of a syndromic presentation of the condition. Here, we report the identification of a variant in *RARG*, the gene encoding the gamma subunit of the RA receptor (RARγ) segregating with the phenotype in this family and describe a dominant-negative mechanism through which it produces KDSM in both humans and a mouse model of the disorder.

## Subjects and methods

**Figure 1:**
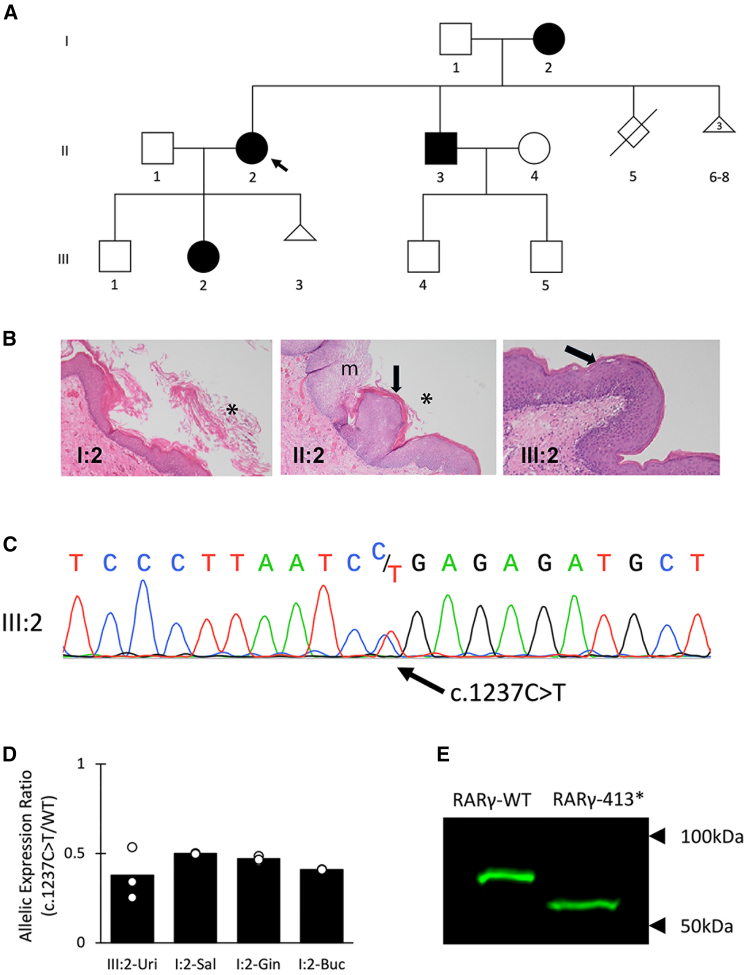
A truncating mutation in *RARG* segregates with a keratinizing desquamative squamous metaplasia (KDSM) phenotype in a three-generation family (A) Pedigree of the family. An arrow denotes the index patient. (B) Histological appearances of the bladder urothelium in I:1, II:2, and III:2. Asterisks denote patches of desquamation, the arrows keratinization, and a region of squamous metaplasia (m). (C) Sanger sequence trace from the genomic DNA of individual III:2 showing the individual heterozygous for the *RARG* NM_000966.6*:*c.1237C>T variant. (D) Transcripts encoding the *RARG* c.1237C>T variant do not undergo nonsense-mediated decay. MiSeq analysis of the relative expression of the mutant and wild-type (WT) alleles in four different samples from two affected individuals. Uri, urinary sediment; Sal, salivary gland; Gin, gingiva; Buc, buccal brushings). Bars represent means calculated from three of technical replicates. (E) Western blot for RARγ in lysate from HEK293FT cells transfected with constructs encoding RARγ-WT and RARγ-413∗.

The clinical details of this previously described family^16^ are summarized in Figure 1A and Table 1. Briefly, three females and one male across three generations developed symptomatic KDSM before adolescence. All four individuals had recurrent urinary tract infections, chronic irritative urinary tract symptoms, and episodic flank pain associated with the passage of keratin debris in the urine. Biopsies of the bladder demonstrated variable combinations of squamous metaplasia, keratinization, and desquamation (Figure 1B). When symptomatic, individuals were treated with intermittent cystoscopic and ureteroscopic debridement. All affected individuals were of mild short stature (−1.1 to −1.8 SD), and all reported dry skin and xerostomia and/or dysphagia. Three of the four affected individuals reported dry eyes requiring daily use of eye drops.

**Table 1:** Demographic information and clinical symptoms and signs in affected individuals

	Patient 1 (II:2)	Patient 2 (III:2)	Patient 3 (I:2)	Patient 4 (II:3)	Totals
Demographics					
Age (years)	48	25	73	38	
Sex	F	F	F	M	
Height (SD) (cm)	151 (−1.8)	156 (−1.1)	154.5 (−1.3)	165 (−1.6)	
Urological (HP:0000079)					
Irritative urinary symptoms	+	+	+	+	4/4
Constant debris in urine	+	+	+	–	3/4
Urethral stricture	–	–	+	+	2/4
Onset of UTIs^a^	Childhood	Childhood	Adolescence	Infancy	
Episodic flank pain	+	+	+	+	4/4
Non-urological					
Dry skin (HP:0000958)	+	+	+	+	4/4
Dry eyes (HP:0001097)	+	+	+	–	3/4
Dry mouth (HP:0000217)	+	–	+	+	3/4
Dysphagia (HP:0002015)	+	+	+	–	3/4
Constipation (HP:0002019)	–	–	+	–	1/4
Vaginal dryness (HP:0031088)	+	–	+	N/A	2/3
Miscarriages	1	0	3	N/A	2/3
Serum vitamin A (200–800 μg/L)	562	617	406	892	

a UTIs, urinary tract infections; HP, human phenotype ontology.

The methods and analytical approach adopted to perform whole-genome analysis on this family are summarized in the supplemental information, as are details of the design of *RARG* expression constructs containing the identified variant alongside other control constructs, the protocols used to assess RARγ signaling and the approach taken to create a mouse model of this condition using CRISPR-Cas9 gene editing in C57BL/6J embryos.

## Results

To identify a genetic cause for KDSM in this family, whole-genome sequencing was performed on three of the four affected individuals (I:2, II:2, III:2), with data analyzed under an autosomal dominant model of inheritance. Variants predicting an alteration to the coding genome were filtered based on rarity in the healthy population (allele count ≤ 2 in gnomAD v.2, *n* = 125,748 exomes and *n* = 15,708 genomes), high or moderate impact (stop-loss, stop-gain, start-loss, frameshift, canonical splice site, missense, in-frame insertion/deletion variants), and segregation with the phenotype before subsequent manual curation of the remaining variants. Thirteen missense variants and one truncating variant (a nonsense variant in *RARG*) were identified after the initial filtering steps. Of the 13 missense variants, 12 were de-prioritized because of the implication of their genes in other distinct Mendelian traits, and expression restricted to non-urogenital tissues. Of the two remaining variants (a missense variant in *SURF6* and a truncating variant in *RARG*), the nonsense variant in *RARG* (NM_000966.6:c.1237C>T) was identified as the top candidate for further characterization. It predicts the creation of a premature stop codon in the final exon to produce a truncated protein lacking 42 C-terminal amino acid residues (NP_000957.1:p.Arg413∗). All four affected individuals were confirmed to be heterozygous for the c.1237C>T variant in *RARG* using Sanger sequencing (Figure 1C). The *RARG* variant is not represented in gnomAD,^17^ ClinVar, or reported in the literature. *RARG* has a probability of being tolerant to loss-of-function variation (pLI score of 0.38 in gnomAD v4.1.0^17^) and a loss-of-function observed/expected upper bound fraction score of 0.604, implying that, if a truncating variant solely confers haploinsufficency to the locus, then it is unlikely to be responsible for the phenotype described here. Notably, to date, no diseases have been associated with this gene.

*RARG* encodes RARγ, one of three subunits for receptors for RA (RARα, RARβ, and RARγ). These subunits pair with RXR subunits to form functional receptors.^1^ *RARG* expression is highest in esophageal mucosa and skin, followed by other squamous epithelia (e.g., vagina and cervix).^18^ *RARG* is strongly expressed in the urogenital tract (median value 35.05 transcripts per million^18^).

To determine whether the premature stop codon created by c.1237C>T confers haploinsufficiency for the allele through nonsense-mediated decay of the *RARG* transcript, MiSeq sequencing was performed on cDNA synthesized from RNA obtained from urinary sediment from individual III:2, and from biopsies of minor salivary glands, gingiva, and buccal brushings from individual I:2. The region flanking the c.1237C>T variant in *RARG* transcripts was amplified from cDNA and sequenced for the presence of the variant. In all four samples, the allelic representation of the variant, as measured by the number of reads with and without the variant, did not differ significantly from 0.5 (Figure 1D). These data indicate that the variant containing transcripts are stable and predict the production of a truncated protein from this allele.

To investigate the stability of truncated p.Arg413∗ RARG protein, wild-type (WT) and truncated (413∗) cDNA *RARG* constructs were cloned into expression plasmids and transfected into HEK293FT cells. Western analysis of cell lysates demonstrated that both variant and control constructs produced protein in comparable amounts (Figure 1E), suggesting that the disease-associated c.1237C>T variant produces a truncated RARγ that is stable, but the effect of the loss of the C-terminal 42 residues on receptor function is unclear.

**Figure 2:**
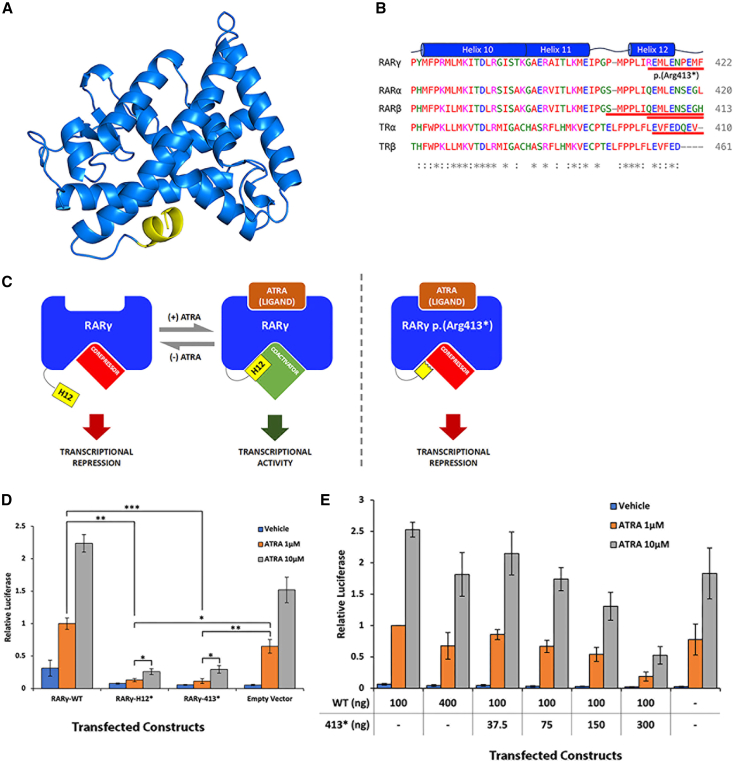
Retinoic acid receptor gamma (RARγ) p.Arg413∗ lacks half of helix 12 and acts in a dominant-negative manner to alter receptor mediated signaling (A) Crystal structure of the ligand binding domain of RARγ^20^ with helix 12 shaded yellow (color edited using PyMOL software). (B) Sequence homology between RARγ (NP_000957.1), paralogous retinoic acid receptors (RARα NP_000955.1 and RARβ NP_000956.2), and two thyroid hormone receptors (TRα NP_955366.1 and TRβ NP_001341641.1). Alignment created using Clustal Omega. Asterisks indicate identical residues, colons indicate residues with similar biophysical properties. Helix locations as defined in Renaud et al.^20^ The p.Arg413∗ truncation removes the C-terminal five amino acid residues from the eight-residue long helix 12 (H12). Disease-causing truncations in RARβ (dbSNP: rs1575553547, rs1701836507) and TRα^23^ are also shown with red underline. (C) Binding of ligand (e.g., all-*trans* retinoic acid; ATRA) to RARγ alters H12 (yellow) conformation, which influences coregulator binding and subsequent transcriptional activity of the receptor. The truncated H12 in RARγ p.Arg413∗ cannot stabilize coactivator binding and instead permits corepressor binding resulting in transcriptional repression regardless of ligand occupancy. (D) RARγ-413∗ exhibits diminished responsiveness to ATRA, resulting in signaling activity comparable with RARγ-H12∗ which lacks the whole of H12. HEK293FT cells were co-transfected with the Cignal RARE reporter and constructs encoding either RARγ-WT, RARγ-H12∗, RARγ-413∗, or empty vector, and subsequently treated with ATRA (1 μM, 10 μM) or vehicle (DMSO). Relative luciferase expression is expressed as a fold response compared with RARG-WT treated with 1 μM ATRA set at 1.0. Error bars show standard deviation. ∗*p* < 0.05, ∗∗*p* < 0.01, ∗∗∗*p* < 0.001; Welch’s *t* test. (E) RARγ-413∗ exerts RARE repression of co-transfected RARγ-WT in a dose-dependent fashion. HEK293FT cells were co-transfected with Cignal RARE reporter and RARγ-WT, RARγ-WT with RARγ-413∗ at the indicated amounts, or with empty vector. Cells were subsequently treated with ATRA (1 μM, 10 μM) or vehicle (DMSO). Relative luciferase expression is expressed as a fold response compared with RARG-WT (100 ng) treated with 1 μM ATRA set at 1.0. Error bars show standard deviation.

RARγ is part of the nuclear receptor superfamily, which share a general structure composed of a DNA-binding domain and a ligand binding domain (LBD).^19^ The LBD in RARs consists of an iterative series of α helices (H1–H12).^20^ The p.Arg413∗ variant found in the family described here removes five C-terminal residues from the eight-residue long H12 (Figures 2A and 2B^8^^,^^21^^,^^22^^,^^23^). Binding of ligand, all*-trans* RA (ATRA), to the receptor stabilizes the conformation in which H12 is tightly packed to the LBD.^20^ In this position, H12 facilitates the binding of coactivators by forming a charge clamp between glutamate on H12 and lysine on H3 (Glu414 and Lys246 in RARγ). This same interaction between H12 and H3 precludes the binding of corepressors and hence further facilitates ligand-mediated signaling. Disengagement of RARγ from ATRA leads to displacement of H12 from its interaction with H3 and facilitates corepressor binding; although, this likely depends on the cellular context.^24^^,^^25^ RARγ p.Arg413∗ has a shortened H12 and lacks the abovementioned critical glutamate residue and therefore may lack both coactivator binding and permit corepressor binding, resulting in constitutive repression regardless of ligand occupancy (Figure 2C).

To assess the hypothesis that this C-terminal truncated RARγ protein exerts a repressive effect on signaling, we examined the effect of the RARγ-413∗ variant on RARγ-sponsored transcriptional activity via a luciferase-based transcription assay. Previous work has shown that the complete loss of H12 in RARγ results in the repression of basal RARE activity from endogenous RARs.^26^ Thus, we compared luciferase activity in HEK293FT cells transfected with a RARγ-413∗ construct to those with RARγ-WT and RARγ-H12∗ (a construct with a truncation beginning at the start of H12 i.e., p.Pro410∗). RARγ-413∗ was functionally comparable with the RARγ-H12∗, with both showing repression of endogenous RARE activation with 1 μM ATRA stimulation compared with the empty vector controls (*p* = 0.007 and *p* = 0.01, respectively; Welch’s *t* test) and transfected RARγ-WT (*p* = 0.0001 and *p* = 0.0019, respectively; Welch’s *t* test) (Figure 2D). There was some release of repression upon stimulation of both RARγ-413∗ and RARγ-H12∗ with higher concentrations of ATRA (Figure 2D).

The reduced signaling activity conferred by the p.Arg413∗ variant could conceivably be explained by insufficiency-mediated or dominant-negative mechanisms. RARγ and RXR subunits heterodimerize to constitute functional RA receptors and incorporation of a dimerization-competent but signaling-incompetent RARγ subunit into receptor dimers could impair signaling through the latter mechanism. As observed above, RARγ-413∗ repressed global endogenous RARE activation, suggesting a likely dominant-negative mechanism. To explore this mechanism directly, activation of the RARE reporter by RARγ-WT was measured in the presence of increasing amounts of RARγ-413∗. At all concentrations of ATRA, RARγ-413∗ exerted RARE repression of co-transfected RARγ-WT, with increasing repression being observed at higher levels of RARγ-413∗ (Figure 2E).

KDSM is typically a sporadic disorder that affects older individuals, most commonly females. To assess the possibility that somatically acquired variants in *RARG* could explain some of these presentations we performed deep sequencing on the urinary sediment of four individuals (three females, one male) undergoing treatment for KDSM. No *RARG* variants were observed (data not shown).

**Figure 3:**
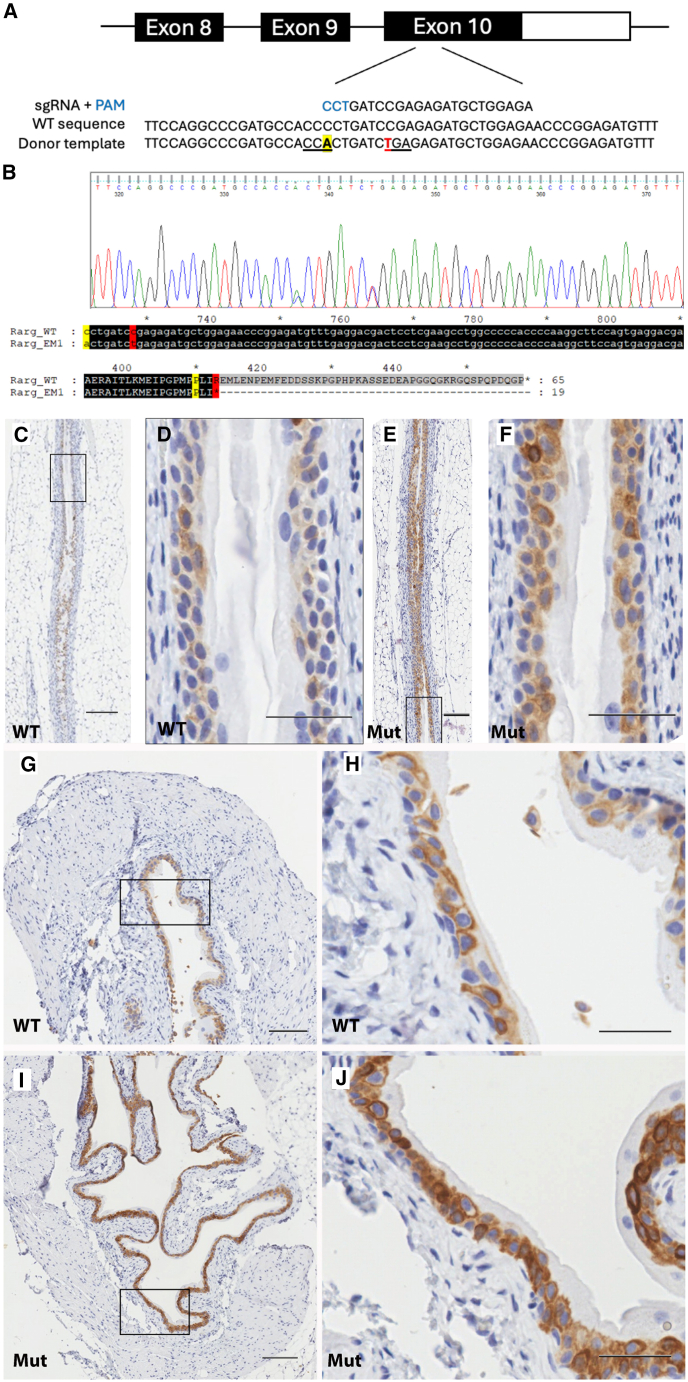
*Rarg*^*Arg413∗/+*^ mice exhibit incipient urothelial metaplasia (A) Schematic of the *Rarg*^*Arg413∗*^ mutation introduced in exon 10 (genome assembly GRCm39m transcript ENSMUST00000043172.15) showing the WT mouse sequence, the gRNA with the PAM in blue and the DNA donor template. The Arg413∗ (CGA > TGA) substitution is marked in red and the silent mutation on Pro410 (CCC > CCA) to mutate the PAM sequence is shown in yellow. (B) Sanger sequencing results from an F1 heterozygous mouse and the resulting cDNA and protein mutant sequences. (C–J) Mice heterozygous for *Rarg*^*Arg413∗*^ demonstrate epithelial metaplasia. Immunohistochemical staining for cytokeratin 10 (CK10), a marker of incipient squamous keratinizing metaplasia^13^^,^^14^ in longitudinal sections of the ureters (C–F) and parasagittal sections of the bladder wall (G–J) from 4-month-old female mice heterozygous for the *Rarg*^*413∗*^ variant. An intensification of CK10 staining in both apical and basal cells of both urothelial surfaces is evident. Boxed regions (C, E, G, and I) refer to magnified views presented in (D), (F), (H), and (J), respectively. Scale bars, 35 μm (D, F, H, and J) and 100 μm (C, E, G, and I).

To generate further evidence that the *RARG* Arg413∗ variant leads to KDSM we engineered C57BL/6J mice carrying this variant (Figure 3). Mice were produced with normal Mendelian ratios and heterozygotes were indistinguishable from their WT littermates in terms of their anthropometry, health, behavior, and fertility. Mice were sacrificed at P7 and P120 and block resections of the urethra, bladder, ureters, and kidneys were obtained, fixed, embedded, sectioned, and immunostained for markers of the urothelium (uroplakin-III [UPIII]) and incipient secondary keratinization of the urothelium (cytokeratin-10 [CK10]).^13^^,^^14^ Histochemical staining of the bladder and ureters of both WT and mutant mice at P7 and P120 demonstrated no frank keratinization of any urothelial surface. At P7 the bladder wall and ureters of both WT and *Rarg*^*Arg413∗/+*^ animals demonstrated strong staining for UPIII and negligible staining for CK10 (data not shown). However, longitudinal sectioning of the ureters from *Rarg*^*Arg413∗/+*^ animals aged to p120 demonstrated much stronger staining for CK10 compared with WT, consistent with epithelial squamous metaplasia (Figure 3). Additionally, CK10 staining of the bladder urothelium in older (P120) animals demonstrated a marked intensification of CK10 staining also indicative of squamous metaplasia.

## Discussion

A lack of vitamin A, the inactive precursor of RA, results in keratinizing squamous metaplasia in the urothelium, as well as in other epithelial surfaces in other organs.^3^ Latterly, RA, likely produced by underlying stromal cells, has been shown to act as both a suppressor of squamous metaplasia but also a promoter of differentiation of the transitional epithelium of the bladder and ureter.^27^^,^^28^ The identity of the signaling pathway by which these effects were mediated was uncertain. Previously, *Rarg* null mice have been shown to develop axial malformations^29^ and notably defects of the Harderian glands (orbital glands for lubrication of the eye) and seminal vesicles. KDSM is generally a sporadic condition with no clear etiology yet described. The family studied here is the second described in the literature.^12^ Our finding of a likely pathogenic variant in *RARG* implicates RA signaling in the development of KDSM, a finding that is congruent with studies on vitamin A deficiency in rats and mice.^3^^,^^13^

Supporting the proposed pathogenicity of the *RARG* variant studied here, disease-causing alleles with similar or equivalent truncations have been identified in the genes encoding other nuclear receptors (Figure 2B). A truncation in RARβ (NP_000956.2:p.Ser398∗) located between H11 and H12 in an individual with syndromic microphthalmia (MCOPS12) was shown to repress the activity of the endogenous WT receptor^8^ and several missense variants affecting H12 (Leu402Pro, Ile403Thr, Leu407Pro) also exhibit the same dominant-negative effect and a truncation of RARβ (Gln404∗) at exactly the analogous residue to the RARγ p.Arg413∗ variant studied here also causes MCOPS12.^8^ Additionally, a truncation in TRα, another nuclear hormone receptor with a similar LBD to RARG, sited one residue C-terminal to the position equivalent to p.Arg413∗ in RARγ (NP_955366.1:p.Glu403∗) causes thyroid hormone resistance through a dominant-negative effect.^23^ Notably variants predicting partial or complete removal of H12 with preservation of preceding protein folds contributing to the rest of the LBD are absent in large databases of genetic variants from otherwise healthy people.^17^

While the urothelial keratinization was the most troubling feature for the individuals in this family, they also shared some extra-urological manifestations including mild short stature and dryness of various epithelial surfaces (ocular, oral, vaginal). Congruent with our findings here, vitamin A deprivation in rats also produces keratinization in the epithelial structures of the salivary glands, respiratory epithelium, uterine glands, prostate and seminal vesicles, conjunctiva, cornea, and lacrimal gland.^3^ In rats, there is evidence of complete cessation of growth in bone, and it has since been demonstrated that RARγ is involved in growth plate function.^29^ In mouse embryogenesis, RARγ expression is localized to cartilage and differentiating squamous keratinizing epithelia irrespective of embryological origin.^30^ These data support the idea of these extra-urological manifestations in the family being part of the syndrome caused by the variant in *RARG*.

No somatic mutations in *RARG* were found in four sporadic KDSM patients. The pathogenesis of sporadic KDSM therefore remains incompletely understood and a range of environmental factors, including nutritional status, could compound with deficient RA signaling to result in expression of this phenotype.

RARγ-Arg413∗ demonstrates reduced responsivity to ATRA, but this effect is lessened at higher concentrations of ATRA. Whether increased levels of ATRA ligand are acting on the truncated receptor to produce a response or maximizing activity from endogenous WT receptor is unclear, but these data suggest there is potential for treatment of the affected individuals in this family with RARγ-specific agonists.

## Data and code availability

The genome sequences used to identify the variant described here are available from the authors upon reasonable request and alignment with the ethical protocols granted for this study. All other datasets included in this published report are publicly available. The variant identified has been submitted to ClinVar, SUB15937138.

## Acknowledgments

This work was supported by the Dean’s Medical Student Research Scholarship from Otago Medical School, CureKids, the Southland Medical Research Foundation Trust, the MRC National Mouse Genetics Network (MC_PC_21044; to S.R.F.T. and K.J.L.) and the 10.13039/501100000272National Institute for Health Research (NIHR) Oxford Biomedical Research Centre (to S.R.F.T.). We are grateful for the excellent support of the Mary Lyon Centre team, especially Sara Wells, Lydia Teboul, Michelle Stewart, James Cleak, Jacqueline Horn, Anju Paudyal, and Lynn Beresford. Histology was performed in the Histology Unit, Research Infrastructure Center at the University of Otago.

## Declaration of interests

The authors declare no competing interests.

## Supplemental information

Document S1. Supplemental materials and methods

Document S2. Article plus supplemental information
