# Supplementary material for: Impaired retinoic acid receptor-γ signaling underlies a heritable form of urothelial keratinizing squamous metaplasia
Source: HGG Adv. 2026 Mar 13;7(2):100590. doi: 10.1016/j.xhgg.2026.100590 (PMC13050057; doi:10.1016/j.xhgg.2026.100590)
Supplement: Document S1. Supplemental materials and methods [file mmc1.pdf]

HGGA, Volume 7

Supplemental information

Impaired retinoic acid receptor-γ signaling underlies a heritable form of urothelial keratinizing squamous metaplasia

Kaya Fukushima, Nicole Avery, Jade Desjardins, Benjamin J. Halliday, Zandra A. Jenkins, Robert Porteous, Tim Morgan, Padmini Parthasarathy, Michael Lau, Michael W. Vincent, Karen J. Liu, Stephen R.F. Twigg, and Stephen P. Robertson

Supplementary Materials and Methods

Human subjects and ethical approval

Individuals with biopsy-proven KDSM from the previously described family [1] (Fig 1A) and sporadic cases were identified through clinician-initiated referral and enrolled under approved ethical protocols MEC/08/08/094 and 13/STH/56 (Health and Disability Ethics Committee, New Zealand). The patients in this manuscript have given written informed consent to publication of their clinical details.

Genome sequencing

Genomic DNA was extracted from whole blood from individuals I:2, II:2, and III:3, and whole genome sequencing (WGS) performed as previously described [2]. Briefly, DNA libraries were prepared using the TruSeq Nano DNA Library Prep kit v2.5 (Illumina), and the paired-end reads were aligned to the reference sequence (GRCh37 assembly) using the Burrows-Wheeler Aligner v0.7.17 with the MEM algorithm [3]. GATK HaplotypeCaller v3.8 was used to call single nucleotide variants/indels and these variants were annotated with gene context information using SnpEff (v4.3S).

Variant confirmation

Genomic DNA was extracted from a saliva sample from individual II:3 according to standard protocols. The AmpliTaq Gold polymerase kit (Applied Biosystems) was used to amplify the region surrounding *RARG* for confirmatory Sanger sequencing.

Transcript analysis

RNA was isolated from urinary debris from individual III:2 and biopsies of minor salivary glands, gingiva, and buccal brushings from individual I:2 using NucleoSpin RNA Plus kit

(Machery Nagel) and treated using the TURBO DNA-free kit (Invitrogen). RNA was converted to cDNA using SuperScript III (Thermo Fisher Scientific) with random primers (Thermo Fisher Scientific) and OligoDT (custom DNA oligos, Merck) oligonucleotides. Primary amplicon polymerase chain reaction (PCR) was carried out with primers, that incorporated Illumina adaptor linkers, within exon 9 and 10 of *RARG* (NM_000966.6) designed to produce a product with >50 base pairs flanking the variant position. Amplification was run to the early log phase, products were purified on Agencourt AMPure XP (Beckman Coulter), and concentration was determined on a Qubit with the Qubit dsDNA HS Assay Kit (Thermo Fisher Scientific). Second-round index PCR (ten cycles) was performed with re-purification as above, before sequencing on a MiSeq instrument (Reagent Nano Kit v2 (500 cycles; Illumina)). Reads were aligned to the reference sequence using the Burrows-Wheeler Aligner v0.7.17 with the MEM algorithm [3], and counts for each base at each position was obtained using IGVtools [4].

Generation of constructs

RARG (NM_000966.6) was amplified from cDNA obtained from the HEK293FT cell line and cloned into pcDNA3.1 with a C-terminal FLAG-twinSTREP tag. The RARγ-413* (NP_000957.1:p.(Arg413*)) and RARγ-H12* (deletion at helix 12; removing amino acids 410-454) variants were produced by PCR mutagenesis using Q5 High-Fidelity DNA Polymerase (New England Biolabs). Cloning utilized the NEBuilder HiFi DNA Assembly kit (New England Biolabs).

In-vitro expression

HEK293FT cells were cultured in DMEM, 10% FCS, penicillin/streptomycin 100 µg/ml at 37 °C with 5% CO_2. Transfection of RARγ-WT or RARγ-413* used Lipofectamine 2000 (Thermo Fisher Scientific). After 24 hours, cells were lysed in RIPA buffer followed by

denaturation in Laemmli sample buffer. Samples were separated on a 10% SDS-PAGE gel and transferred to a nitrocellulose membrane on a Trans-Blot Turbo (Bio-Rad). Membranes were incubated with mouse anti-FLAG (Merck, F1804, 1:7,000 dilution) and IRDye secondary antibodies (LI-COR Biosciences, IRDYE 800CW Goat anti-Mouse, 1:25,000 dilution). Membranes were visualised on the Odyssey CLx and images were analysed using Image Studio software (LI-COR Biosciences).

Luciferase assay

Relative RARγ transcriptional activation was assayed using the Cignal retinoic acid response element (RARE) reporter assay kit (QIAGEN). HEK293FT cells were co-transfected with Cignal RARE reporter and either RARγ-WT, RARγ-H12*, RARγ-413* or empty vector, such that RARγ expression levels were optimized to equalize relative expression of RARγ-WT, RARγ-H12* and RARγ-413*. For assessing the dominant negative effects of the RARγ-413* variant, constructs encoding RARγ-WT and RARγ-413* were co-transfected at various concentrations, in conjunction with the Cignal RARE reporter. Cells were treated with all-*trans* retinoic acid (ATRA; 1 μM, 10 μM) or vehicle (DMSO) 24h post transfection and incubated for a further 16 hours. Firefly and renilla luciferase activities were measured using the Dual-Glo Luciferase Assay System as per the manufacturers protocol (Promega). Relative firefly luciferase expression was normalized to renilla luciferase. Data represents the averages of three independent experiments with each sample carried out in triplicate. Protein levels were assayed from replicate wells.

Somatic mutation analysis

DNA was isolated from urinary debris from four patients with sporadic KDSM using Promega Wizard Genomic DNA Purification Kit. The AmpliTaq Gold polymerase kit

(Applied Biosystems) was used to individually amplify the protein-coding exons in *RARG* for Sanger sequencing. The region involving the 50 base pairs flanking our identified variant position in Exon 10 of *RARG* (NM_000966.6) was also sequenced using the MiSeq system described for the transcript analysis, to assess for mosaicism.

Gene editing in mouse

Generation of *Rarg<em1H>* (MGI allele ID: 8253168. MGI strain ID: 8253169) was performed by introduction of a point mutation encoding p.(Arg413*) into the mouse *Rarg* locus (this amino acid is equivalent in human and mouse) using CRISPR/Cas9. C57BL/6J embryos were electroporated at the 1-cell stage with 90:10 deactivated Cas9:wildtype Cas9 protein, sgRNA (TCTCCAGCATCTCTCGGATC) and a single stranded donor oligonucleotide (5'-GCACTTCTGCTCTCCCAATCCTCTTCGTGTCTCTCTGTAGGAGCAGAAAGGGCTATAACCCTGAAGATGGAGATTCCAGGCCCGATGCCACC**a**CTGATC**t**GAGAGATGCTGGAGAACCCGGAGATGTTTGAGGACGACTCCTCGAAGCCTGGCCCCCACCCCAAGGCTTCCAGTGAGGACGAAGCTCCAGGGGGCCAGGG-3'; note the lower case a and t represent a base change to the PAM to prevent re-cutting, and the c>t variant encoding Arg413*, respectively). Deactivated Cas9 was used to prevent excess cutting of both alleles. Cas9 protein, sgRNAs and ssODNs were diluted and mixed in electroporation buffer (EB; Gibco Opti-MEM I Reduced Serum Media – (Thermo Fisher Scientific)) to the working concentrations of 650 ng/µl, 130 ng/µl total and 400 ng/µl, respectively. Embryos were electroporated using the following conditions: 40 V, 3.5 ms pulse length, 50 ms pulse interval, 4 pulses (NEPA21 Type II – (NEPA Gene)). Electroporated embryos were re-implanted in CD1 pseudo-pregnant females. Host females were allowed to litter and rear F_0 progeny. Screening of founders and offspring of positive founders was performed using

genomic DNA extracted from ear clip biopsies, PCR amplification (primers: R413*F, 5'-CTGAGAGTTGGGCACATAGTTGT-3' and R413*R, 5'-TGGTCTCTAGTGTTCCTGTTTGC-3') and Sanger sequencing. A Taqman assay was designed to screen for additional integrations of the donor sequence; none were detected in animals taken forward to establish the colony.

Mouse breeding and preparation of tissue

All animals were produced and housed at the Mary Lyon Centre (MLC), MRC Harwell Institute under specific opportunistic pathogen-free (SOPF) conditions, in individually ventilated cages adhering to environmental conditions as outlined in the Home Office Code of Practice. All animal studies were carried out under the Animals (Scientific Procedures) Act 1986 Amendment Regulations 2012. Animal work was approved by the Institutional Ethical Review Committee and performed in accordance with the UK Home Office Project Licenses PP0015552, PP5106593 and PP9404876. $Rarg^{Arg413*/+}$ mice were bred and maintained under a 12-hour light/12-hour dark cycle and fed *ad libitum* with Teklad diet (Inotiv) before sacrifice at 4 months of age. The urogenital tract was dissected, formalin-fixed and paraffin-embedded before sectioning for immunostaining.

Mouse immunostaining

Antibodies used: anti-uroplakinIII (ProGen cat# 690108S) diluted 1:1000 in Leica Bond diluent (Leica Biosystems AR9352) and anti-cytokeratin10 (Invitrogen MA5-42858) at 1:400 dilution in BioCare Renoir Red diluent (BioCare Medical PD904L). All slides were processed for IHC on a Leica BondRx Autostainer using the Leica Polymer Refine Detection kit (Leica Biosystems DS9800). Heat induced epitope retrieval was done using a citrate buffer (Leica Biosystems AR9961) for 20 minutes at 95 °C.

References

1. Avery N, Fukushima K, Guan G, et al. Familial aggregation of keratinising desquamative squamous metaplasia in the urinary tract. *BJU Int*. 2024;133(S3):15-17. doi:10.1111/BJU.16160
2. Mi J, Parthasarathy P, Halliday BJ, et al. Deletion of exon 1 in amer1 in osteopathia striata with cranial sclerosis. *Genes (Basel)*. 2020;11(12):1-7. doi:10.3390/GENES11121439
3. Li H, Durbin R. Fast and accurate short read alignment with Burrows–Wheeler transform. *bioinformatics*. 2009;25(14):1754-1760.
4. Robinson JT, Thorvaldsdóttir H, Winckler W, et al. Integrative genomics viewer. *Nat Biotechnol*. 2011;29(1):24-26.
