# Supplementary material for: Impaired retinoic acid receptor-γ signaling underlies a heritable form of urothelial keratinizing squamous metaplasia
Source: HGG Adv. 2026 Mar 13;7(2):100590. doi: 10.1016/j.xhgg.2026.100590 (PMC13050057; doi:10.1016/j.xhgg.2026.100590)
Supplement: Document S2. Article plus supplemental information [file mmc2.pdf]

HGG Advances

BRIEF COMMUNICATION

Impaired retinoic acid receptor-γ signaling underlies a heritable form of urothelial keratinizing squamous metaplasia

Kaya Fukushima,[1] Nicole Avery,[2] Jade Desjardins,[3,4] Benjamin J. Halliday,[1] Zandra A. Jenkins,[1] Robert Porteous,[5] Tim Morgan,[1] Padmini Parthasarathy,[1] Michael Lau,[6] Michael W. Vincent,[7] Karen J. Liu,[3,4] Stephen R.F. Twigg,[4,8] and Stephen P. Robertson[1,9,*]

Summary

Keratinizing desquamative squamous metaplasia (KDSM) of the urinary tract is typically a sporadic condition with unclear etiology and treatment options. It is characterized by either a focal or widespread transition of normal urothelium of the bladder and ureters to a stratified squamous keratinizing epithelium. Four individuals from three generations of a single family were ascertained with a likely autosomal dominant form of syndromic KDSM. Whole-genome sequencing was performed on three affected individuals and a truncating variant (*RARG* NM_000966.6:c.1237C>T; NP_000957.1:p.Arg413*) in the gene encoding retinoic acid receptor gamma (RARγ) was identified to be segregating with the phenotype. The truncating variant does not destabilize the transcript or protein produced from this allele but instead predicts the loss of half of helix 12 of RARγ, leading to reduced responsiveness of the receptor to all-*trans* retinoic acid via a dominant-negative mechanism. Mice heterozygous for the variant demonstrated upregulation of cytokeratin-10 in the bladder and ureteric epithelium consistent with keratinizing squamous metaplasia of the urothelium. The implicated dominant-negative mechanism reduces retinoic acid signaling via heterodimeric receptors that incorporate the variant γ subunit and indicates that this condition may be addressable with high-dose retinoic acid receptor agonists.

Introduction

Retinoic acid (RA) signaling is widely deployed across development.[1,2] A deficiency of vitamin A, the precursor for RA, results in disorders of epithelia[3] and reflective of this role in epithelial development and maintenance, RA analogs are used therapeutically to treat disorders of epithelia by influencing cellular differentiation.[4–6]

RA exerts its biochemical effects through binding to cytoplasmic receptors that are composed of heterodimers of either one of RARα, RARβ, or RARγ in conjunction with one of three RXR isoforms. Engagement of RA with these receptors results in the translocation of the complex to the nucleus and the activation of various transcriptional programs by binding to specific sequences (RA response elements [RAREs]) within gene promoters and enhancers.[7] Diminished or insufficient signaling via RA receptors lead to a variety of disorders including a monogenic disorder characterized by microphthalmia (MCOPS12, OMIM: 615524), caused by truncating variants in *RARB*.[8]

Keratinizing desquamative squamous metaplasia (KDSM) of the urinary tract is a rare, typically sporadic condition that is characterized by the transition of normal urothelium to a stratified squamous keratinizing epithelium.[9] Keratinized cellular debris typically accumulates over time within the bladder and ureter manifesting clinically with lower urinary tract symptoms including urgency, frequency, ureteric obstruction, and occasionally hematuria.[9] Most reported cases have been sporadic with no clear etiology identified. Chronic inflammation, irritation, and micronutrient deficiencies are all possible factors contributing to the pathogenesis of the condition.[10,11] Genetic factors have also been suggested based on the occurrence of KDSM in a single family, consistent with autosomal dominant inheritance of the trait.[9,12] Several reports have described instances where vitamin A supplementation was successful in treating KDSM in the context of vitamin A deficiency.[10,11] Consistent with a possible link between KDSM and RA signaling, vitamin A deficiency in murine models reproduces a phenotype similar to KDSM.[3,13–15]

Recently, we reported a family of four individuals (three females, one male) from three generations affected by a syndromic form of KDSM.[16] In addition to urothelial metaplasia, they also reported dry mouth, dry eyes, and manifested mild short stature, suggestive of a syndromic presentation of the condition. Here, we report the identification of a variant in *RARG*, the gene encoding the

[1]Department of Paediatrics and Child Health, Dunedin School of Medicine, University of Otago, Dunedin 9016, New Zealand; [2]Department of Urology, Dunedin Hospital, Dunedin 9016, New Zealand; [3]Centre for Craniofacial and Regenerative Biology, King's College London, SE1 9RT London, UK; [4]MRC National Mouse Genetics Network, Congenital Anomalies Cluster, Harwell, OX11 ORD Oxfordshire, UK; [5]Department of Pathology, Dunedin School of Medicine, University of Otago, Dunedin 9016, New Zealand; [6]Awanui Laboratories, Dunedin 9016, New Zealand; [7]Department of Urology, Southland Hospital, Invercargill 9812, New Zealand; [8]MRC Weatherall Institute of Molecular Medicine, John Radcliffe Hospital, University of Oxford, OX3 9DS Oxford, UK
[9]Lead contact
*Correspondence: stephen.robertson@otago.ac.nz
https://doi.org/10.1016/j.xhgg.2026.100590.
© 2026 The Author(s). Published by Elsevier Inc. on behalf of American Society of Human Genetics.
This is an open access article under the CC BY license (http://creativecommons.org/licenses/by/4.0/).

gamma subunit of the RA receptor (RARγ) segregating with the phenotype in this family and describe a dominant-negative mechanism through which it produces KDSM in both humans and a mouse model of the disorder.

Subjects and methods

The clinical details of this previously described family[16] are summarized in Figure 1A and Table 1. Briefly, three females and one male across three generations developed symptomatic KDSM before adolescence. All four individuals had recurrent urinary tract infections, chronic irritative urinary tract symptoms, and episodic flank pain associated with the passage of keratin debris in the urine. Biopsies of the bladder demonstrated variable combinations of squamous metaplasia, keratinization, and desquamation (Figure 1B). When symptomatic, individuals were treated with intermittent cystoscopic and ureteroscopic debridement. All affected individuals were of mild short stature (−1.1 to −1.8 SD), and all reported dry skin and xerostomia and/or dysphagia. Three of the four affected individuals reported dry eyes requiring daily use of eye drops.

The methods and analytical approach adopted to perform whole-genome analysis on this family are summarized in the supplemental information, as are details of the design of *RARG* expression constructs containing the identified variant alongside other control constructs, the protocols used to assess RARγ signaling and the approach taken to create a mouse model of this condition using CRISPR-Cas9 gene editing in C57BL/6J embryos.

Results

To identify a genetic cause for KDSM in this family, whole-genome sequencing was performed on three of the four affected individuals (I:2, II:2, III:2), with data analyzed under an autosomal dominant model of inheritance. Variants predicting an alteration to the coding genome were filtered based on rarity in the healthy population (allele count ≤ 2 in gnomAD v.2, $n = 125{,}748$ exomes and $n = 15{,}708$ genomes), high or moderate impact (stop-loss, stop-gain, start-loss, frameshift, canonical splice site, missense, in-frame insertion/deletion variants), and segregation with the phenotype before subsequent manual curation of the remaining variants. Thirteen missense variants and one truncating variant (a nonsense variant in *RARG*) were identified after the initial filtering steps. Of the 13 missense variants, 12 were de-prioritized because of the implication of their genes in other distinct Mendelian traits, and expression restricted to non-urogenital tissues. Of the two remaining variants (a missense variant in *SURF6* and a truncating variant in *RARG*), the nonsense variant in *RARG* (NM_000966.6:c.1237C>T) was identified as the top candidate for further characterization. It predicts the creation of a premature stop codon in the final exon to produce a truncated protein lacking 42 C-terminal amino acid residues (NP_000957.1:p.Arg413*). All four affected individuals were confirmed to be heterozygous for the c.1237C>T variant in *RARG* using Sanger sequencing (Figure 1C). The *RARG* variant is not represented in gnomAD,[17] ClinVar, or reported in the literature. *RARG* has a probability of being tolerant to loss-of-function variation (pLI score of 0.38 in gnomAD v4.1.0[17]) and a loss-of-function observed/expected upper bound fraction score of 0.604, implying that, if a truncating variant solely confers haploinsufficency to the locus, then it is unlikely to be responsible for the phenotype described here. Notably, to date, no diseases have been associated with this gene.

RARG encodes RARγ, one of three subunits for receptors for RA (RARα, RARβ, and RARγ). These subunits pair with RXR subunits to form functional receptors.[1] *RARG* expression is highest in esophageal mucosa and skin, followed by other squamous epithelia (e.g., vagina and cervix).[18] *RARG* is strongly expressed in the urogenital tract (median value 35.05 transcripts per million[18]).

To determine whether the premature stop codon created by c.1237C>T confers haploinsufficiency for the allele through nonsense-mediated decay of the *RARG* transcript, MiSeq sequencing was performed on cDNA synthesized from RNA obtained from urinary sediment from individual III:2, and from biopsies of minor salivary glands, gingiva, and buccal brushings from individual I:2. The region flanking the c.1237C>T variant in *RARG* transcripts was amplified from cDNA and sequenced for the presence of the variant. In all four samples, the allelic representation of the variant, as measured by the number of reads with and without the variant, did not differ significantly from 0.5 (Figure 1D). These data indicate that the variant containing transcripts are stable and predict the production of a truncated protein from this allele.

To investigate the stability of truncated p.Arg413* RARG protein, wild-type (WT) and truncated (413*) cDNA *RARG* constructs were cloned into expression plasmids and transfected into HEK293FT cells. Western analysis of cell lysates demonstrated that both variant and control constructs produced protein in comparable amounts (Figure 1E), suggesting that the disease-associated c.1237C>T variant produces a truncated RARγ that is stable, but the effect of the loss of the C-terminal 42 residues on receptor function is unclear.

RARγ is part of the nuclear receptor superfamily, which share a general structure composed of a DNA-binding domain and a ligand binding domain (LBD).[19] The LBD in RARs consists of an iterative series of α helices (H1–H12).[20] The p.Arg413* variant found in the family described here removes five C-terminal residues from the eight-residue long H12 (Figures 2A and 2B[8,21–23]). Binding of ligand, all-*trans* RA (ATRA), to the receptor stabilizes the conformation in which H12 is tightly packed to the LBD.[20] In this position, H12 facilitates the binding of coactivators by forming a charge clamp between glutamate on H12 and lysine on H3 (Glu414 and Lys246 in RARγ). This same interaction between H12 and H3 precludes the binding of corepressors and hence further facilitates

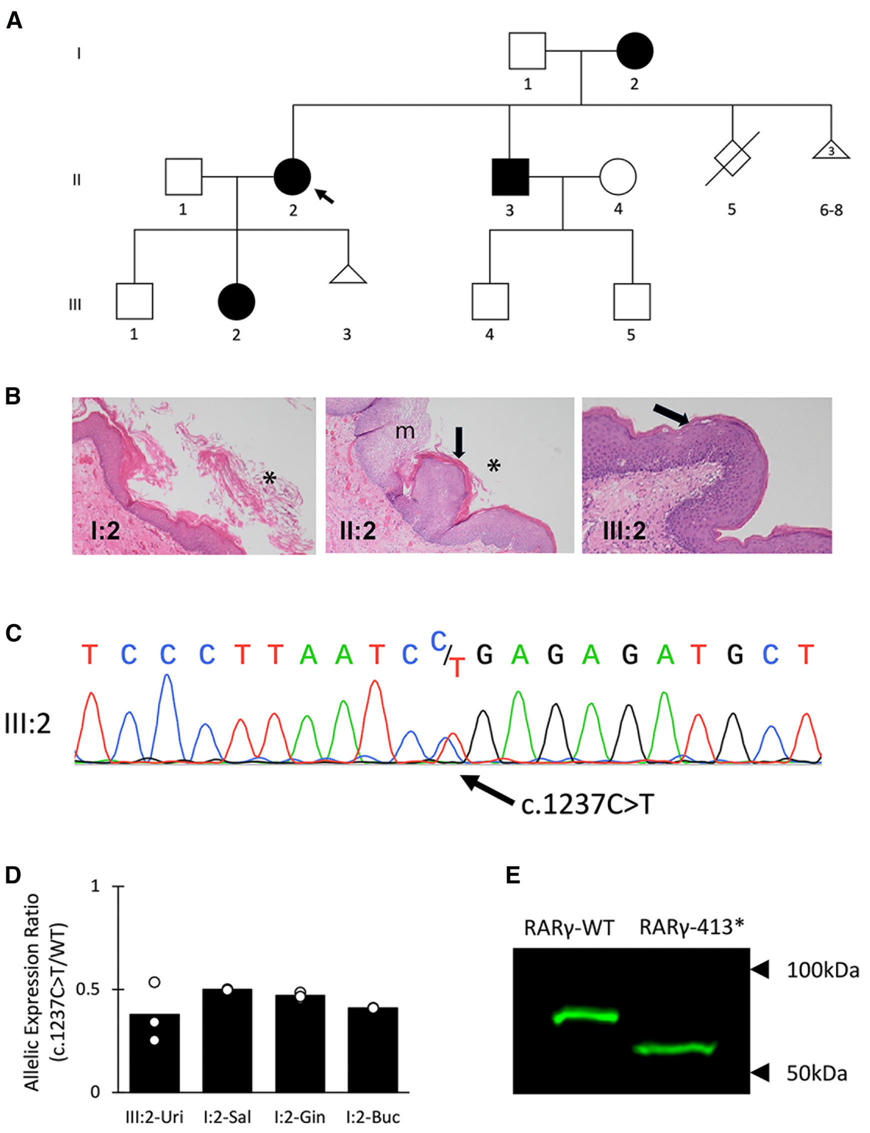

Figure 1. A truncating mutation in *RARG* segregates with a keratinizing desquamative squamous metaplasia (KDSM) phenotype in a three-generation family

(A) Pedigree of the family. An arrow denotes the index patient.

(B) Histological appearances of the bladder urothelium in I:1, II:2, and III:2. Asterisks denote patches of desquamation, the arrows keratinization, and a region of squamous metaplasia (m).

(C) Sanger sequence trace from the genomic DNA of individual III:2 showing the individual heterozygous for the *RARG* NM_000966.6:c.1237C>T variant.

(D) Transcripts encoding the *RARG* c.1237C>T variant do not undergo nonsense-mediated decay. MiSeq analysis of the relative expression of the mutant and wild-type (WT) alleles in four different samples from two affected individuals. Uri, urinary sediment; Sal, salivary gland; Gin, gingiva; Buc, buccal brushings). Bars represent means calculated from three of technical replicates.

(E) Western blot for RARγ in lysate from HEK293FT cells transfected with constructs encoding RARγ-WT and RARγ-413*.

ligand-mediated signaling. Disengagement of RARγ from ATRA leads to displacement of H12 from its interaction with H3 and facilitates corepressor binding; although, this likely depends on the cellular context.[24,25] RARγ p.Arg413* has a shortened H12 and lacks the abovementioned critical glutamate residue and therefore may lack both coactivator binding and permit corepressor binding, resulting in constitutive repression regardless of ligand occupancy (Figure 2C).

To assess the hypothesis that this C-terminal truncated RARγ protein exerts a repressive effect on signaling, we examined the effect of the RARγ-413* variant on RARγ-sponsored transcriptional activity via a luciferase-based transcription assay. Previous work has shown that the complete loss of H12 in RARγ results in the repression of basal RARE activity from endogenous RARs.[26] Thus, we compared luciferase activity in HEK293FT cells transfected with a RARγ-413* construct to those with RARγ-WT and RARγ-H12* (a construct with a truncation beginning at the start of H12 i.e., p.Pro410*). RARγ-413* was functionally comparable with the RARγ-H12*, with both showing repression of endogenous RARE activation with 1 μM ATRA stimulation compared with the empty vector controls ($p = 0.007$ and $p = 0.01$, respectively; Welch's *t* test) and transfected RARγ-WT ($p = 0.0001$ and $p = 0.0019$, respectively; Welch's *t* test) (Figure 2D). There was some release of repression upon stimulation of both RARγ-413* and RARγ-H12* with higher concentrations of ATRA (Figure 2D).

The reduced signaling activity conferred by the p.Arg413* variant could conceivably be explained by insufficiency-mediated or dominant-negative mechanisms. RARγ and RXR subunits heterodimerize to constitute functional RA receptors and incorporation of a dimerization-competent but signaling-incompetent RARγ subunit into receptor dimers could impair signaling through the latter mechanism. As observed above, RARγ-413* repressed global endogenous RARE activation, suggesting a likely dominant-negative mechanism. To explore this mechanism directly, activation of the RARE reporter by RARγ-WT was measured in the presence of increasing amounts of RARγ-413*. At all concentrations of ATRA, RARγ-413* exerted RARE repression of co-transfected RARγ-WT, with increasing repression being observed at higher levels of RARγ-413* (Figure 2E).

Table 1. Demographic information and clinical symptoms and signs in affected individuals

	Patient 1 (II:2)	Patient 2 (III:2)	Patient 3 (I:2)	Patient 4 (II:3)	Totals
Demographics					
Age (years)	48	25	73	38	
Sex	F	F	F	M	
Height (SD) (cm)	151 (−1.8)	156 (−1.1)	154.5 (−1.3)	165 (−1.6)	
Urological (HP:0000079)					
Irritative urinary symptoms	+	+	+	+	4/4
Constant debris in urine	+	+	+	–	3/4
Urethral stricture	–	–	+	+	2/4
Onset of UTIs[a]	Childhood	Childhood	Adolescence	Infancy	
Episodic flank pain	+	+	+	+	4/4
Non-urological					
Dry skin (HP:0000958)	+	+	+	+	4/4
Dry eyes (HP:0001097)	+	+	+	–	3/4
Dry mouth (HP:0000217)	+	–	+	+	3/4
Dysphagia (HP:0002015)	+	+	+	–	3/4
Constipation (HP:0002019)	–	–	+	–	1/4
Vaginal dryness (HP:0031088)	+	–	+	N/A	2/3
Miscarriages	1	0	3	N/A	2/3
Serum vitamin A (200–800 μg/L)	562	617	406	892	

[a]UTIs, urinary tract infections; HP, human phenotype ontology.

KDSM is typically a sporadic disorder that affects older individuals, most commonly females. To assess the possibility that somatically acquired variants in *RARG* could explain some of these presentations we performed deep sequencing on the urinary sediment of four individuals (three females, one male) undergoing treatment for KDSM. No *RARG* variants were observed (data not shown).

To generate further evidence that the *RARG* Arg413* variant leads to KDSM we engineered C57BL/6J mice carrying this variant (Figure 3). Mice were produced with normal Mendelian ratios and heterozygotes were indistinguishable from their WT littermates in terms of their anthropometry, health, behavior, and fertility. Mice were sacrificed at P7 and P120 and block resections of the urethra, bladder, ureters, and kidneys were obtained, fixed, embedded, sectioned, and immunostained for markers of the urothelium (uroplakin-III [UPIII]) and incipient secondary keratinization of the urothelium (cytokeratin-10 [CK10]).[13,14] Histochemical staining of the bladder and ureters of both WT and mutant mice at P7 and P120 demonstrated no frank keratinization of any urothelial surface. At P7 the bladder wall and ureters of both WT and $Rarg^{Arg413*/+}$ animals demonstrated strong staining for UPIII and negligible staining for CK10 (data not shown). However, longitudinal sectioning of the ureters from $Rarg^{Arg413*/+}$ animals aged to p120 demonstrated much stronger staining for CK10 compared with WT, consistent with epithelial squamous metaplasia (Figure 3). Additionally, CK10 staining of the bladder urothelium in older (P120) animals demonstrated a marked intensification of CK10 staining also indicative of squamous metaplasia.

Discussion

A lack of vitamin A, the inactive precursor of RA, results in keratinizing squamous metaplasia in the urothelium, as well as in other epithelial surfaces in other organs.[3] Latterly, RA, likely produced by underlying stromal cells, has been shown to act as both a suppressor of squamous metaplasia but also a promoter of differentiation of the transitional epithelium of the bladder and ureter.[27,28] The identity of the signaling pathway by which these effects were mediated was uncertain. Previously, *Rarg* null mice have been shown to develop axial malformations[29] and notably defects of the Harderian glands (orbital glands for lubrication of the eye) and seminal vesicles. KDSM is generally a sporadic condition with no clear etiology yet described. The family studied here is the second described in the literature.[12] Our finding of a likely pathogenic variant in *RARG* implicates RA signaling in the development of KDSM, a finding that is congruent with studies on vitamin A deficiency in rats and mice.[3,13]

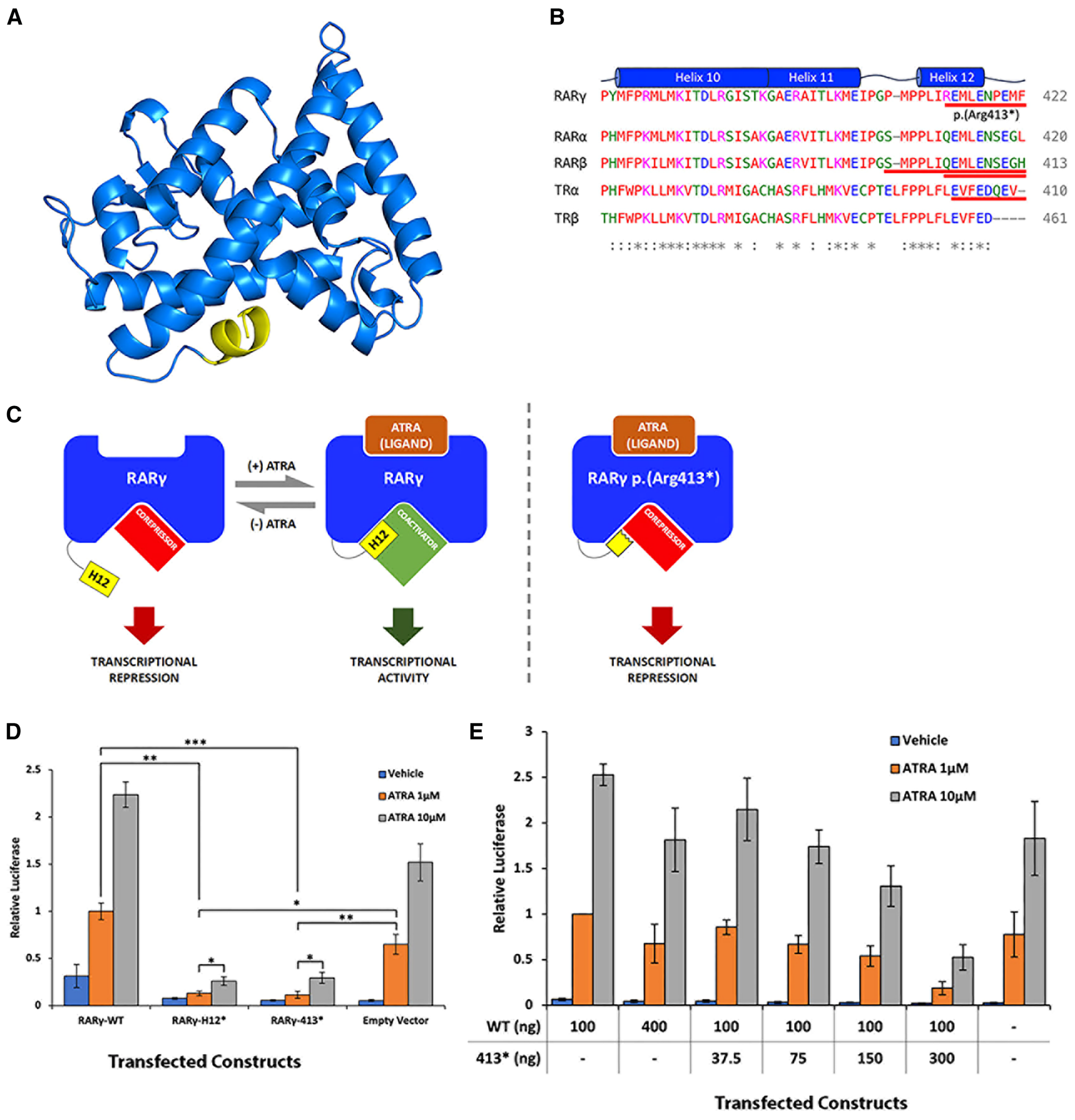

Figure 2. Retinoic acid receptor gamma (RARγ) p.Arg413* lacks half of helix 12 and acts in a dominant-negative manner to alter receptor mediated signaling

(A) Crystal structure of the ligand binding domain of RARγ[20] with helix 12 shaded yellow (color edited using PyMOL software).

(B) Sequence homology between RARγ (NP_000957.1), paralogous retinoic acid receptors (RARα NP_000955.1 and RARβ NP_000956.2), and two thyroid hormone receptors (TRα NP_955366.1 and TRβ NP_001341641.1). Alignment created using Clustal Omega. Asterisks indicate identical residues, colons indicate residues with similar biophysical properties. Helix locations as defined in Renaud et al.[20] The p.Arg413* truncation removes the C-terminal five amino acid residues from the eight-residue long helix 12 (H12). Disease-causing truncations in RARβ (dbSNP: rs1575553547, rs1701836507) and TRα[23] are also shown with red underline.

(C) Binding of ligand (e.g., all-*trans* retinoic acid; ATRA) to RARγ alters H12 (yellow) conformation, which influences coregulator binding and subsequent transcriptional activity of the receptor. The truncated H12 in RARγ p.Arg413* cannot stabilize coactivator binding and instead permits corepressor binding resulting in transcriptional repression regardless of ligand occupancy.

(D) RARγ-413* exhibits diminished responsiveness to ATRA, resulting in signaling activity comparable with RARγ-H12* which lacks the whole of H12. HEK293FT cells were co-transfected with the Cignal RARE reporter and constructs encoding either RARγ-WT, RARγ-H12*, RARγ-413*, or empty vector, and subsequently treated with ATRA (1 μM, 10 μM) or vehicle (DMSO). Relative luciferase expression is expressed as a fold response compared with RARG-WT treated with 1 μM ATRA set at 1.0. Error bars show standard deviation. *$p < 0.05$, **$p < 0.01$, ***$p < 0.001$; Welch's t test.

(E) RARγ-413* exerts RARE repression of co-transfected RARγ-WT in a dose-dependent fashion. HEK293FT cells were co-transfected with Cignal RARE reporter and RARγ-WT, RARγ-WT with RARγ-413* at the indicated amounts, or with empty vector. Cells were subsequently treated with ATRA (1 μM, 10 μM) or vehicle (DMSO). Relative luciferase expression is expressed as a fold response compared with RARG-WT (100 ng) treated with 1 μM ATRA set at 1.0. Error bars show standard deviation.

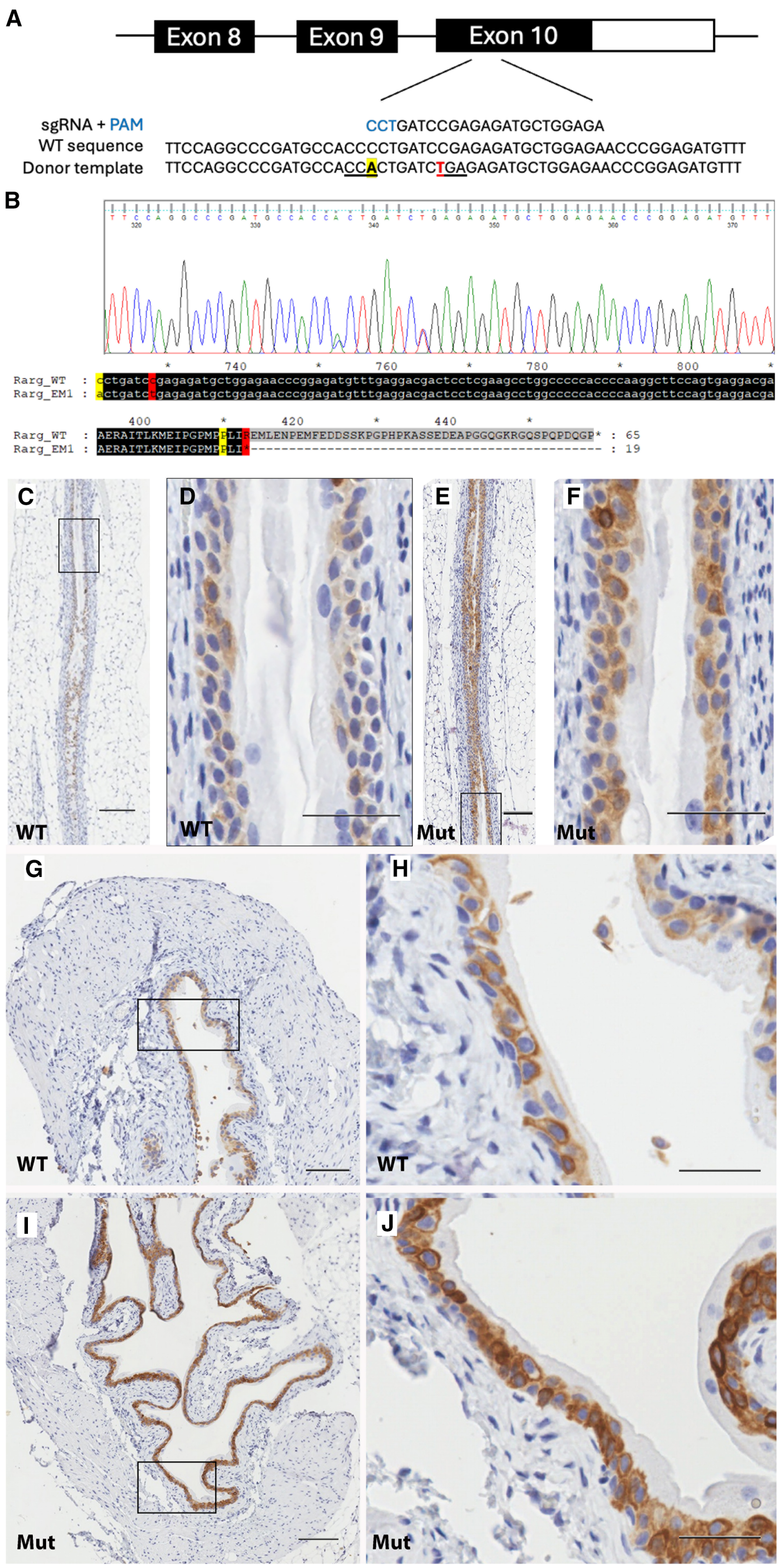

Figure 3. $Rarg^{Arg413*/+}$ mice exhibit incipient urothelial metaplasia

(A) Schematic of the $Rarg^{Arg413*}$ mutation introduced in exon 10 (genome assembly GRCm39m transcript ENSMUST00000043172.15) showing the WT mouse sequence, the gRNA with the PAM in blue and the DNA donor template. The Arg413* (CGA > TGA) substitution is marked in red and the silent mutation on Pro410 (CCC > CCA) to mutate the PAM sequence is shown in yellow.

(B) Sanger sequencing results from an F1 heterozygous mouse and the resulting cDNA and protein mutant sequences.

(C–J) Mice heterozygous for $Rarg^{Arg413*}$ demonstrate epithelial metaplasia. Immunohistochemical staining for cytokeratin 10 (CK10), a marker of incipient squamous keratinizing metaplasia[13,14] in longitudinal sections of the ureters (C–F) and parasagittal sections of the bladder wall (G–J) from 4-month-old female mice heterozygous for the $Rarg^{413*}$ variant. An intensification of CK10 staining in both apical and basal cells of both urothelial surfaces is evident. Boxed regions (C, E, G, and I) refer to magnified views presented in (D), (F), (H), and (J), respectively. Scale bars, 35 μm (D, F, H, and J) and 100 μm (C, E, G, and I).

Supporting the proposed pathogenicity of the *RARG* variant studied here, disease-causing alleles with similar or equivalent truncations have been identified in the genes encoding other nuclear receptors (Figure 2B). A truncation in RARβ (NP_000956.2:p.Ser398*) located between H11 and H12 in an individual with syndromic microphthalmia (MCOPS12) was shown to repress the activity of the endogenous WT receptor[8] and several missense variants affecting H12 (Leu402Pro, Ile403Thr, Leu407Pro) also exhibit the same dominant-negative effect and a truncation of RARβ (Gln404*) at exactly the analogous residue to the RARγ p.Arg413* variant studied here also causes MCOPS12.[8] Additionally, a truncation in TRα, another nuclear hormone receptor with a similar LBD to RARG, sited one residue C-terminal to the position equivalent to p.Arg413* in RARγ (NP_955366.1: p.Glu403*) causes thyroid hormone resistance through a dominant-negative effect.[23] Notably variants predicting partial or complete removal of H12 with preservation of preceding protein folds contributing to the rest of the LBD are absent in large databases of genetic variants from otherwise healthy people.[17]

While the urothelial keratinization was the most troubling feature for the individuals in this family, they also shared some extra-urological manifestations including mild short stature and dryness of various epithelial surfaces (ocular, oral, vaginal). Congruent with our findings here, vitamin A deprivation in rats also produces keratinization in the epithelial structures of the salivary glands, respiratory epithelium, uterine glands, prostate and

seminal vesicles, conjunctiva, cornea, and lacrimal gland.[3] In rats, there is evidence of complete cessation of growth in bone, and it has since been demonstrated that RARγ is involved in growth plate function.[29] In mouse embryogenesis, RARγ expression is localized to cartilage and differentiating squamous keratinizing epithelia irrespective of embryological origin.[30] These data support the idea of these extra-urological manifestations in the family being part of the syndrome caused by the variant in *RARG*.

No somatic mutations in *RARG* were found in four sporadic KDSM patients. The pathogenesis of sporadic KDSM therefore remains incompletely understood and a range of environmental factors, including nutritional status, could compound with deficient RA signaling to result in expression of this phenotype.

RARγ-Arg413* demonstrates reduced responsivity to ATRA, but this effect is lessened at higher concentrations of ATRA. Whether increased levels of ATRA ligand are acting on the truncated receptor to produce a response or maximizing activity from endogenous WT receptor is unclear, but these data suggest there is potential for treatment of the affected individuals in this family with RARγ-specific agonists.

Data and code availability

The genome sequences used to identify the variant described here are available from the authors upon reasonable request and alignment with the ethical protocols granted for this study. All other datasets included in this published report are publicly available. The variant identified has been submitted to ClinVar, SUB15937138.

Acknowledgments

This work was supported by the Dean's Medical Student Research Scholarship from Otago Medical School, CureKids, the Southland Medical Research Foundation Trust, the MRC National Mouse Genetics Network (MC_PC_21044; to S.R.F.T. and K.J.L.) and the National Institute for Health Research (NIHR) Oxford Biomedical Research Centre (to S.R.F.T.). We are grateful for the excellent support of the Mary Lyon Centre team, especially Sara Wells, Lydia Teboul, Michelle Stewart, James Cleak, Jacqueline Horn, Anju Paudyal, and Lynn Beresford. Histology was performed in the Histology Unit, Research Infrastructure Center at the University of Otago.

Declaration of interests

The authors declare no competing interests.

Supplemental information

Supplemental information can be found online at https://doi.org/10.1016/j.xhgg.2026.100590.

Received: January 15, 2026
Accepted: March 10, 2026

References

1. Ghyselinck, N.B., and Duester, G. (2019). Retinoic acid signaling pathways. Development *146*, dev167502.
2. Wu, D., Khan, F.A., Zhang, K., Pandupuspitasari, N.S., Negara, W., Guan, K., Sun, F., and Huang, C. (2024). Retinoic acid signaling in development and differentiation commitment and its regulatory topology. Chem. Biol. Interact. *387*, 110773.
3. Wolbach, S.B., and Howe, P.R. (1925). Tissue changes following deprivation of fat-soluble A vitamin. J. Exp. Med. *42*, 753–777.
4. Fogagnolo, P., De Cilla, S., Alkabes, M., Sabella, P., and Rossetti, L. (2021). A Review of Topical and Systemic Vitamin Supplementation in Ocular Surface Diseases. Nutrients *13*, 1998.
5. Motamedi, M., Chehade, A., Sanghera, R., and Grewal, P. (2022). A Clinician's Guide to Topical Retinoids. J. Cutan. Med. Surg. *26*, 71–78.
6. Samarawickrama, C., Chew, S., and Watson, S. (2015). Retinoic acid and the ocular surface. Surv. Ophthalmol. *60*, 183–195.
7. Di Masi, A., Leboffe, L., De Marinis, E., Pagano, F., Cicconi, L., Rochette-Egly, C., Lo-Coco, F., Ascenzi, P., and Nervi, C. (2015). Retinoic acid receptors: from molecular mechanisms to cancer therapy. Mol. Aspects Med. *41*, 1–115.
8. Caron, V., Chassaing, N., Ragge, N., Boschann, F., Ngu, A.M.H., Meloche, E., Chorfi, S., Lakhani, S.A., Ji, W., Steiner, L., et al. (2023). Clinical and functional heterogeneity associated with the disruption of retinoic acid receptor beta. Genet. Med. *25*, 100856.
9. Ahmad, I., Barnetson, R.J., and Krishna, N.S. (2008). Keratinizing squamous metaplasia of the bladder: a review. Urol. Int. *81*, 247–251.
10. Finta, M.K., Dhaliwal, G., Albin, O.R., Ghani, K.R., and Houchens, N. (2024). Flipping the Switch. N. Engl. J. Med. *390*, 456–462.
11. Bhandari, R., Muhammed, J.M., Gurung, P., and Ekladious, A. (2024). Vitamin A Treatment for Recurrent Urinary Tract Infections in a Post-Roux-en-Y Gastric Bypass Patient: A Case Report: Case Report. American Journal of Medical and Clinical Research & Reviews *3*, 1–6.
12. Mueller, S.C., Thueroff, J.W., and Rumpelt, H.J. (1987). Urothelial leukoplakia: new aspects of etiology and therapy. J. Urol. *137*, 979–983.
13. Liang, F.X., Bosland, M.C., Huang, H., Romih, R., Baptiste, S., Deng, F.M., Wu, X.R., Shapiro, E., and Sun, T.T. (2005). Cellular basis of urothelial squamous metaplasia: roles of lineage heterogeneity and cell replacement. J. Cell Biol. *171*, 835–844.
14. Gijbels, M.J., van der Ham, F., van Bennekum, A.M., Hendriks, H.F., and Roholl, P.J. (1992). Alterations in cytokeratin expression precede histological changes in epithelia of vitamin A-deficient rats. Cell Tissue Res. *268*, 197–203.
15. Molloy, C.J., and Laskin, J.D. (1988). Effect of retinoid deficiency on keratin expression in mouse bladder. Exp. Mol. Pathol. *49*, 128–140.

16. Avery, N., Fukushima, K., Guan, G., Praganta, J., Rich, A., Vincent, M., and Robertson, S. (2024). Familial aggregation of keratinising desquamative squamous metaplasia in the urinary tract. BJU Int. *133*, 15–17.
17. Karczewski, K.J., Francioli, L.C., Tiao, G., Cummings, B.B., Alföldi, J., Wang, Q., Collins, R.L., Laricchia, K.M., Ganna, A., Birnbaum, D.P., et al. (2020). The mutational constraint spectrum quantified from variation in 141,456 humans. Nature *581*, 434–443.
18. (2025). GTEx Portal. https://www.gtexportal.org/home/.
19. Sar, P. (2023). Receptor Endocytosis and Signalling in Health and Disease-Part B. Nucl. Recept.: Structure and function *222*, 209.
20. Renaud, J.P., Rochel, N., Ruff, M., Vivat, V., Chambon, P., Gronemeyer, H., and Moras, D. (1995). Crystal structure of the RAR-γ ligand-binding domain bound to all-trans retinoic acid. Nature *378*, 681–689.
21. Klaholz, B.P., Mitschler, A., and Moras, D. (2000). Structural basis for isotype selectivity of the human retinoic acid nuclear receptor. J. Mol. Biol. *302*, 155–170.
22. Moll, R., Divo, M., and Langbein, L. (2008). The human keratins: Biology and pathology. Histochem. Cell Biol. *129*, 705–733.
23. Bochukova, E., Schoenmakers, N., Agostini, M., Schoenmakers, E., Rajanayagam, O., Keogh, J.M., Henning, E., Reinemund, J., Gevers, E., Sarri, M., et al. (2012). A mutation in the thyroid hormone receptor alpha gene. N. Engl. J. Med. *366*, 243–249.
24. Fischer, A., and Smieško, M. (2019). Ligand pathways in nuclear receptors. J. Chem. Inf. Model. *59*, 3100–3109.
25. Farboud, B., Hauksdottir, H., Wu, Y., and Privalsky, M.L. (2003). Isotype-Restricted Corepressor Recruitment: a Constitutively Closed Helix 12 Conformation in Retinoic Acid Receptors β and γ Interferes with Corepressor Recruitment and Prevents Transcriptional Repression. Mol. Cell Biol. *23*, 2844–2858.
26. Farboud, B., and Privalsky, M.L. (2004). Retinoic acid receptor-α is stabilized in a repressive state by its C-terminal, isotype-specific F domain. Mol. Endocrinol. *18*, 2839–2853.
27. Gandhi, D., Molotkov, A., Batourina, E., Schneider, K., Dan, H., Reiley, M., Laufer, E., Metzger, D., Liang, F., Liao, Y., et al. (2013). Retinoid signaling in progenitors controls specification and regeneration of the urothelium. Dev. Cell *26*, 469–482.
28. Wiessner, G.B., Plumber, S.A., Xiang, T., and Mendelsohn, C.L. (2022). Development, regeneration and tumorigenesis of the urothelium. Development *149*, dev198184.
29. Lohnes, D., Kastner, P., Dierich, A., Mark, M., LeMeur, M., and Chambon, P. (1993). Function of retinoic acid receptor gamma in the mouse. Cell *73*, 643–658.
30. Ruberte, E., Dolle, P., Krust, A., Zelent, A., Morriss-Kay, G., and Chambon, P. (1990). Specific spatial and temporal distribution of retinoic acid receptor gamma transcripts during mouse embryogenesis. Development *108*, 213–222.

HGGA, Volume 7

Supplemental information

Impaired retinoic acid receptor-γ signaling underlies a heritable form of urothelial keratinizing squamous metaplasia

Kaya Fukushima, Nicole Avery, Jade Desjardins, Benjamin J. Halliday, Zandra A. Jenkins, Robert Porteous, Tim Morgan, Padmini Parthasarathy, Michael Lau, Michael W. Vincent, Karen J. Liu, Stephen R.F. Twigg, and Stephen P. Robertson

Supplementary Materials and Methods

Human subjects and ethical approval

Individuals with biopsy-proven KDSM from the previously described family [1] (Fig 1A) and sporadic cases were identified through clinician-initiated referral and enrolled under approved ethical protocols MEC/08/08/094 and 13/STH/56 (Health and Disability Ethics Committee, New Zealand). The patients in this manuscript have given written informed consent to publication of their clinical details.

Genome sequencing

Genomic DNA was extracted from whole blood from individuals I:2, II:2, and III:3, and whole genome sequencing (WGS) performed as previously described [2]. Briefly, DNA libraries were prepared using the TruSeq Nano DNA Library Prep kit v2.5 (Illumina), and the paired-end reads were aligned to the reference sequence (GRCh37 assembly) using the Burrows-Wheeler Aligner v0.7.17 with the MEM algorithm [3]. GATK HaplotypeCaller v3.8 was used to call single nucleotide variants/indels and these variants were annotated with gene context information using SnpEff (v4.3S).

Variant confirmation

Genomic DNA was extracted from a saliva sample from individual II:3 according to standard protocols. The AmpliTaq Gold polymerase kit (Applied Biosystems) was used to amplify the region surrounding *RARG* for confirmatory Sanger sequencing.

Transcript analysis

RNA was isolated from urinary debris from individual III:2 and biopsies of minor salivary glands, gingiva, and buccal brushings from individual I:2 using NucleoSpin RNA Plus kit

(Machery Nagel) and treated using the TURBO DNA-free kit (Invitrogen). RNA was converted to cDNA using SuperScript III (Thermo Fisher Scientific) with random primers (Thermo Fisher Scientific) and OligoDT (custom DNA oligos, Merck) oligonucleotides. Primary amplicon polymerase chain reaction (PCR) was carried out with primers, that incorporated Illumina adaptor linkers, within exon 9 and 10 of *RARG* (NM_000966.6) designed to produce a product with >50 base pairs flanking the variant position. Amplification was run to the early log phase, products were purified on Agencourt AMPure XP (Beckman Coulter), and concentration was determined on a Qubit with the Qubit dsDNA HS Assay Kit (Thermo Fisher Scientific). Second-round index PCR (ten cycles) was performed with re-purification as above, before sequencing on a MiSeq instrument (Reagent Nano Kit v2 (500 cycles; Illumina)). Reads were aligned to the reference sequence using the Burrows-Wheeler Aligner v0.7.17 with the MEM algorithm [3], and counts for each base at each position was obtained using IGVtools [4].

Generation of constructs

RARG (NM_000966.6) was amplified from cDNA obtained from the HEK293FT cell line and cloned into pcDNA3.1 with a C-terminal FLAG-twinSTREP tag. The RARγ-413* (NP_000957.1:p.(Arg413*)) and RARγ-H12* (deletion at helix 12; removing amino acids 410-454) variants were produced by PCR mutagenesis using Q5 High-Fidelity DNA Polymerase (New England Biolabs). Cloning utilized the NEBuilder HiFi DNA Assembly kit (New England Biolabs).

In-vitro expression

HEK293FT cells were cultured in DMEM, 10% FCS, penicillin/streptomycin 100 µg/ml at 37 °C with 5% CO_2. Transfection of RARγ-WT or RARγ-413* used Lipofectamine 2000 (Thermo Fisher Scientific). After 24 hours, cells were lysed in RIPA buffer followed by

denaturation in Laemmli sample buffer. Samples were separated on a 10% SDS-PAGE gel and transferred to a nitrocellulose membrane on a Trans-Blot Turbo (Bio-Rad). Membranes were incubated with mouse anti-FLAG (Merck, F1804, 1:7,000 dilution) and IRDye secondary antibodies (LI-COR Biosciences, IRDYE 800CW Goat anti-Mouse, 1:25,000 dilution). Membranes were visualised on the Odyssey CLx and images were analysed using Image Studio software (LI-COR Biosciences).

Luciferase assay

Relative RARγ transcriptional activation was assayed using the Cignal retinoic acid response element (RARE) reporter assay kit (QIAGEN). HEK293FT cells were co-transfected with Cignal RARE reporter and either RARγ-WT, RARγ-H12*, RARγ-413* or empty vector, such that RARγ expression levels were optimized to equalize relative expression of RARγ-WT, RARγ-H12* and RARγ-413*. For assessing the dominant negative effects of the RARγ-413* variant, constructs encoding RARγ-WT and RARγ-413* were co-transfected at various concentrations, in conjunction with the Cignal RARE reporter. Cells were treated with all-*trans* retinoic acid (ATRA; 1 μM, 10 μM) or vehicle (DMSO) 24h post transfection and incubated for a further 16 hours. Firefly and renilla luciferase activities were measured using the Dual-Glo Luciferase Assay System as per the manufacturers protocol (Promega). Relative firefly luciferase expression was normalized to renilla luciferase. Data represents the averages of three independent experiments with each sample carried out in triplicate. Protein levels were assayed from replicate wells.

Somatic mutation analysis

DNA was isolated from urinary debris from four patients with sporadic KDSM using Promega Wizard Genomic DNA Purification Kit. The AmpliTaq Gold polymerase kit

(Applied Biosystems) was used to individually amplify the protein-coding exons in *RARG* for Sanger sequencing. The region involving the 50 base pairs flanking our identified variant position in Exon 10 of *RARG* (NM_000966.6) was also sequenced using the MiSeq system described for the transcript analysis, to assess for mosaicism.

Gene editing in mouse

Generation of *Rarg<em1H>* (MGI allele ID: 8253168. MGI strain ID: 8253169) was performed by introduction of a point mutation encoding p.(Arg413*) into the mouse *Rarg* locus (this amino acid is equivalent in human and mouse) using CRISPR/Cas9. C57BL/6J embryos were electroporated at the 1-cell stage with 90:10 deactivated Cas9:wildtype Cas9 protein, sgRNA (TCTCCAGCATCTCTCGGATC) and a single stranded donor oligonucleotide (5'-GCACTTCTGCTCTCCCAATCCTCTTCGTGTCTCTCTGTAGGAGCAGAAAGGGCTATAACCCTGAAGATGGAGATTCCAGGCCCGATGCCACC**a**CTGATC**t**GAGAGATGCTGGAGAACCCGGAGATGTTTGAGGACGACTCCTCGAAGCCTGGCCCCCACCCCAAGGCTTCCAGTGAGGACGAAGCTCCAGGGGGCCAGGG-3'; note the lower case a and t represent a base change to the PAM to prevent re-cutting, and the c>t variant encoding Arg413*, respectively). Deactivated Cas9 was used to prevent excess cutting of both alleles. Cas9 protein, sgRNAs and ssODNs were diluted and mixed in electroporation buffer (EB; Gibco Opti-MEM I Reduced Serum Media – (Thermo Fisher Scientific)) to the working concentrations of 650 ng/µl, 130 ng/µl total and 400 ng/µl, respectively. Embryos were electroporated using the following conditions: 40 V, 3.5 ms pulse length, 50 ms pulse interval, 4 pulses (NEPA21 Type II – (NEPA Gene)). Electroporated embryos were re-implanted in CD1 pseudo-pregnant females. Host females were allowed to litter and rear F_0 progeny. Screening of founders and offspring of positive founders was performed using

genomic DNA extracted from ear clip biopsies, PCR amplification (primers: R413*F, 5'-CTGAGAGTTGGGCACATAGTTGT-3' and R413*R, 5'-TGGTCTCTAGTGTTCCTGTTTGC-3') and Sanger sequencing. A Taqman assay was designed to screen for additional integrations of the donor sequence; none were detected in animals taken forward to establish the colony.

Mouse breeding and preparation of tissue

All animals were produced and housed at the Mary Lyon Centre (MLC), MRC Harwell Institute under specific opportunistic pathogen-free (SOPF) conditions, in individually ventilated cages adhering to environmental conditions as outlined in the Home Office Code of Practice. All animal studies were carried out under the Animals (Scientific Procedures) Act 1986 Amendment Regulations 2012. Animal work was approved by the Institutional Ethical Review Committee and performed in accordance with the UK Home Office Project Licenses PP0015552, PP5106593 and PP9404876. $Rarg^{Arg413*/+}$ mice were bred and maintained under a 12-hour light/12-hour dark cycle and fed *ad libitum* with Teklad diet (Inotiv) before sacrifice at 4 months of age. The urogenital tract was dissected, formalin-fixed and paraffin-embedded before sectioning for immunostaining.

Mouse immunostaining

Antibodies used: anti-uroplakinIII (ProGen cat# 690108S) diluted 1:1000 in Leica Bond diluent (Leica Biosystems AR9352) and anti-cytokeratin10 (Invitrogen MA5-42858) at 1:400 dilution in BioCare Renoir Red diluent (BioCare Medical PD904L). All slides were processed for IHC on a Leica BondRx Autostainer using the Leica Polymer Refine Detection kit (Leica Biosystems DS9800). Heat induced epitope retrieval was done using a citrate buffer (Leica Biosystems AR9961) for 20 minutes at 95 °C.

References

1. Avery N, Fukushima K, Guan G, et al. Familial aggregation of keratinising desquamative squamous metaplasia in the urinary tract. *BJU Int*. 2024;133(S3):15-17. doi:10.1111/BJU.16160
2. Mi J, Parthasarathy P, Halliday BJ, et al. Deletion of exon 1 in amer1 in osteopathia striata with cranial sclerosis. *Genes (Basel)*. 2020;11(12):1-7. doi:10.3390/GENES11121439
3. Li H, Durbin R. Fast and accurate short read alignment with Burrows–Wheeler transform. *bioinformatics*. 2009;25(14):1754-1760.
4. Robinson JT, Thorvaldsdóttir H, Winckler W, et al. Integrative genomics viewer. *Nat Biotechnol*. 2011;29(1):24-26.
